# The versatile utility of cysteine as a target for cancer treatment

**DOI:** 10.3389/fonc.2022.997919

**Published:** 2023-01-19

**Authors:** Jin-Young Min, Kyung-Soo Chun, Do-Hee Kim

**Affiliations:** ^1^ Department of Chemistry, Kyonggi University, Suwon, Gyeonggi-do, Republic of Korea; ^2^ College of Pharmacy, Keimyung University, Daegu, Republic of Korea

**Keywords:** cysteine, cancer, resistance, chemotherapy, post-translational modification

## Abstract

Owing to its unique nucleophilicity, cysteine is an attractive sulfhydryl-containing proteinogenic amino acid. It is also utilized in various metabolic pathways and redox homeostasis, as it is used for the component of major endogenous antioxidant glutathione and the generation of sulfur-containing biomolecules. In addition, cysteine is the most nucleophilic amino acid of proteins and can react with endogenous or exogenous electrophiles which can result in the formation of covalent bonds, which can alter the cellular states and functions. Moreover, post-translational modifications of cysteines trigger redox signaling and affect the three-dimensional protein structure. Protein phosphorylation mediated by kinases and phosphatases play a key role in cellular signaling that regulates many physiological and pathological processes, and consequently, the modification of cysteine regulates its activities. The modification of cysteine residues in proteins is critically important for the design of novel types of pharmacological agents. Therefore, in cancer metabolism and cancer cell survival, cysteine plays an essential role in redox regulation of cellular status and protein function. This review summarizes the diverse regulatory mechanisms of cysteine bound to or free from proteins in cancer. Furthermore, it can enhance the comprehension of the role of cysteine in tumor biology which can help in the development of novel effective cancer therapies.

## Introduction

**Figure 1:**
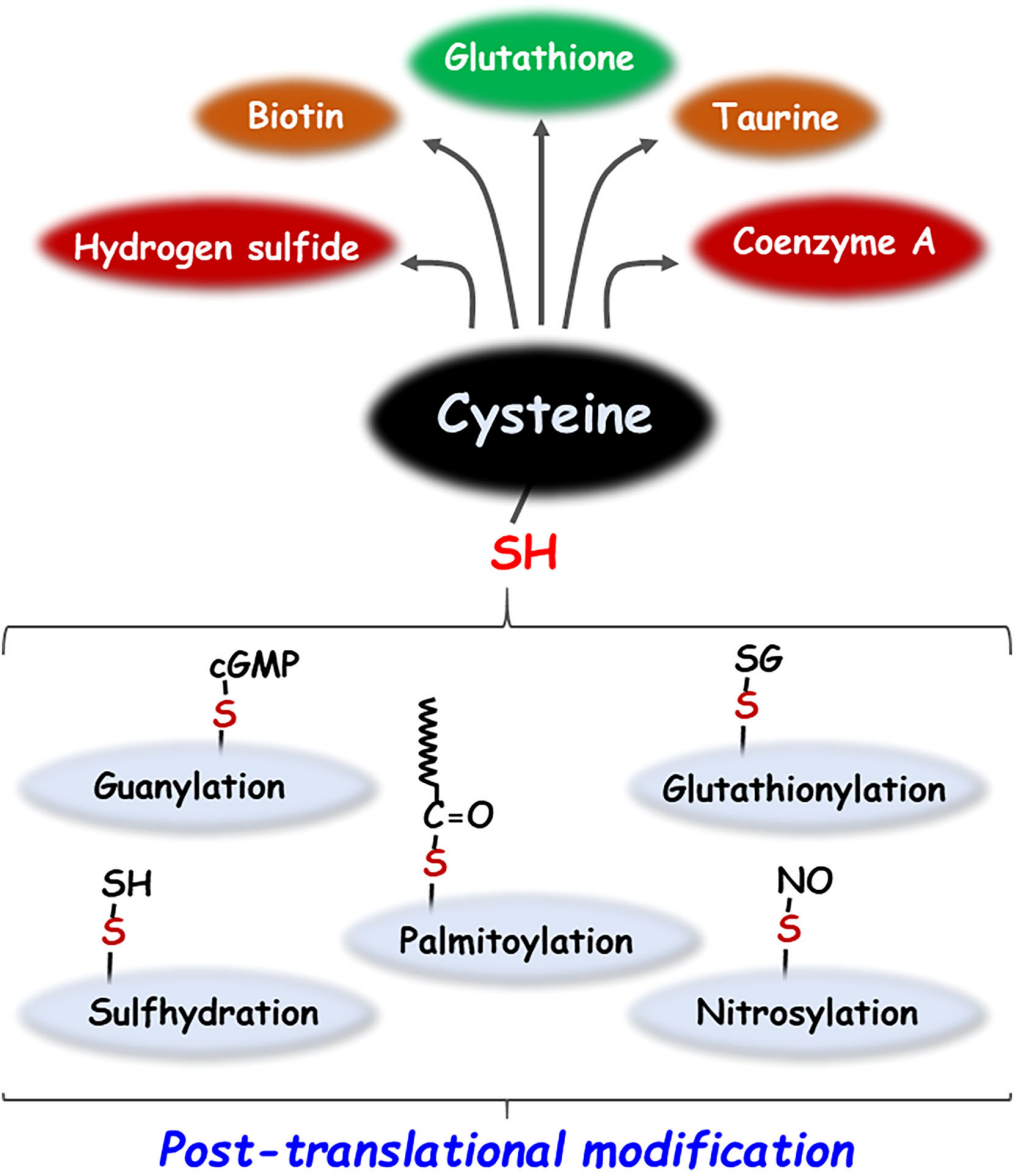
Metabolic products and post-translational modifications of cysteine. Cysteine generates sulfur-containing molecules such as glutathione, taurine, biotin, coenzyme A and hydrogen sulfide by various metabolic pathways. In addition, cysteine undergoes a variety of post-translational modifications including palmitoylation, glutathionylation, guanylation, nitrosylation, and sulfhydration.

Cysteine, a sulfhydryl-containing proteinogenic amino acid essential for the human body, is employed in a variety of metabolic pathways, such as the regulation of reduced glutathione (GSH), a major endogenous antioxidant molecule and the generation of sulfur-containing biomolecules such as hydrogen sulfide (H_2_S), taurine, coenzyme A and biotin (1, 2). In addition, the reactive thiol (-SH) group of cysteine residues in proteins can undergo various post-translational modifications (PTMs) such as palmitoylation, glutathionylation, guanylation, cysteinylation, nitrosylation, and sulfhydration (**Figure 1**) (2). PTMs play a key role in various biological processes by affecting the structure, reactivity, stability, and function of the protein, thereby modulating a broad range of biological processes (3). Moreover, endogenous or exogenous electrophilic molecules can be covalently modified with the nucleophilic cysteine, which consequently alters the cellular states and functions. Some pharmacological agents that exploit the modification of cysteine residues present in many intracellular proteins affect the biological activity, thus exerting their drug effects (4). This review investigates the multifaceted role of cysteine bound to or free from proteins in cancer biology research.

## Cysteine metabolism in cancer and tumor microenvironment

It is increasingly noted that altered levels of amino acids including cysteine in the tumor microenvironment, by uptake or processing of both intracellular and extracellular regions, have emerged as potential markers of tumor progression. Cysteine may interplay with the tumor microenvironment and cancer cell metabolism which affect the metastatic potential and drug resistance (5). The extracellular compartments are highly oxidizing, whereas cells generally maintain a reducing environment in the cytosol.Cysteine mainly exists as cystine, the oxidized dimer form of cysteine, in the extracellular space. Both forms of cysteine and cystine can be imported into cells through specific transporters, and amino acids exist as cysteine in a reducing environment. The cystine/cysteine redox cycle is characterized by a slight increase in the intracellular cysteine levels and exceedingly high extracellular cysteine concentrations, which efficiently protects the cells from oxidative stress-induced cell death (6). Tumors interact with the surrounding microenvironment and organs through the lymphatic network, composed of a complex mixture of cells, including tumor cells, fibroblasts and immune cells. Cystine is transported into stromal cells and converted to cysteine that supports glutathione (GSH) synthesis and secretion into the tumor microenvironment (7). Enhanced cysteine transport and its metabolism enable cancer cells to adapt to a challenging tumor microenvironment and acquire chemoresistance. This section discusses the role of cellular signaling molecules and proteins in regulating cysteine metabolism in cancer development and drug resistance.

### xCT/SLC7A11

**Figure 2:**
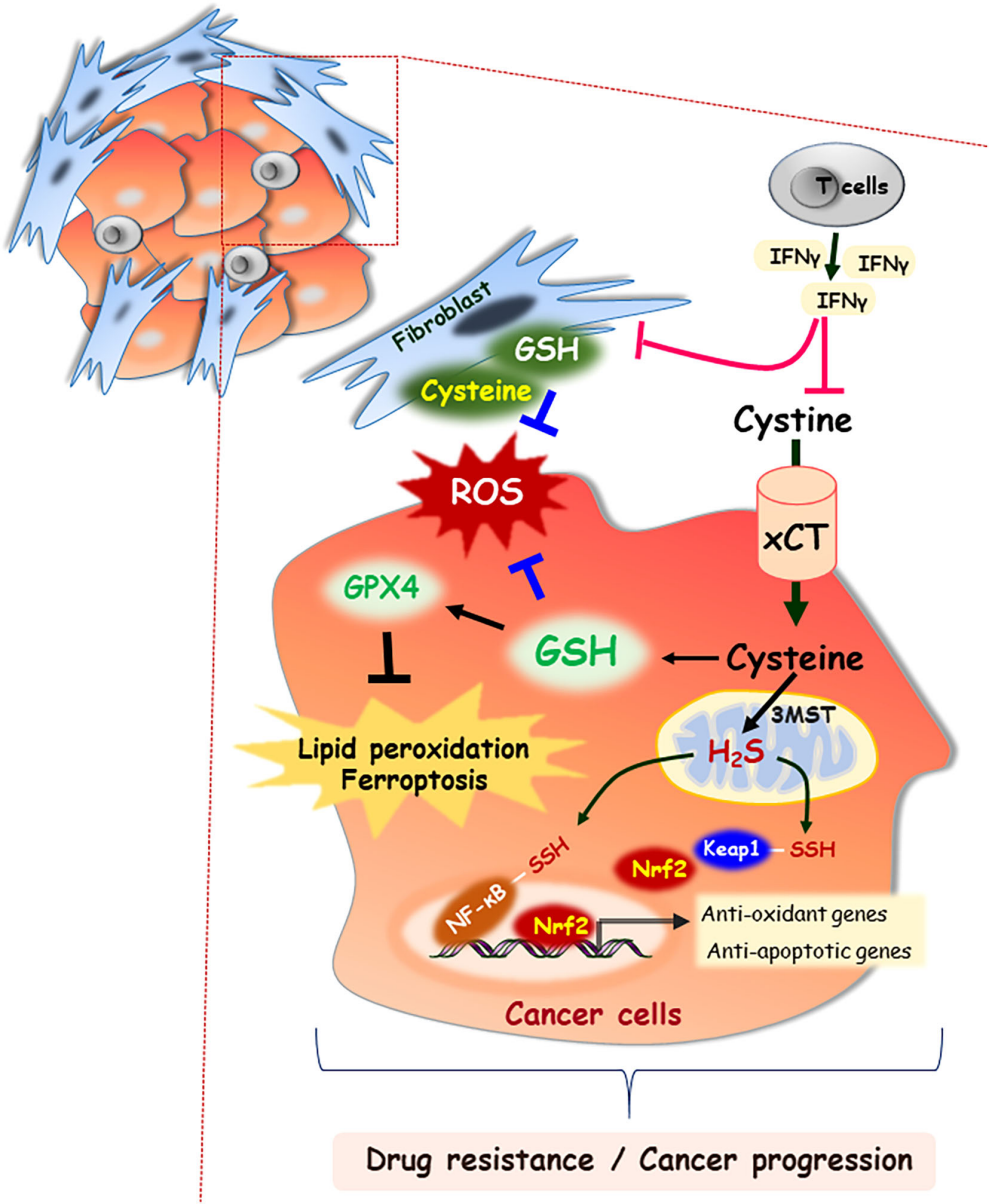
Cysteine as an important building block for cell-cell communication in tumor microenvironment. In the extracellular space, cysteine mainly exists as the oxidized dimer form, cystine. Both forms of cysteine and cystine can be imported into cells through specific transporters, xCT. GSH is synthesized from cysteine, which functions in concert to detoxify ROS. GSH acts as an essential cofactor for GPX4 enzymatic activity, which results in blocking lipid peroxidation and ferroptosis. Stromal fibroblast-derived cysteine and GSH also scavenge the ROS in tumor microenvironment, which contribute to cancer progression. However, IFN-γ derived from CD8^+^ T cells attenuates drug resistance through downregulation of xCT. In addition, 3MST resident in mitochondria generates H_2_S, and then it induces the NF-κB activation by persulfidation of its subunit or nuclear localization of Nrf2 *via* persulfidation of Keap1.

System x_c_^-^ transporter is a heterodimer consisting of the disulfide-linked light (xCT/SLC7A11) and heavy (CD98/SLC3A2) chain subunits. Cystine/glutamate antiporter solute carrier family 7 member 11 (SLC7A11) is a major transporter regulating cysteine in tumor cells and is induced in response to a variety of stimuli, such as oxidative stress and electrophilic compounds (8). Lin et al. reported that SLC7A11 is widely expressed in multiple human cancers, and its up-regulation is correlated with poor survival outcomes in patients with breast cancer, prostate cancer, and papillary thyroid carcinoma (9). In prostate cancer, xCT protein expression is positively associated with invasion and metastasis by affecting the redox status of the tumor microenvironment. Zhong et al. reported that altering the extracellular cysteine/cystine ratio by xCT knockdown inhibits prostate cancer cell invasion (10). In addition, oncogenic KRAS-mutant cancer cells maintain the induction of *xCT* transcription, which enhance the GSH levels and protect tumor cells against oxidative stress (11). Moreover, cysteine and glutathione released from stromal fibroblasts in the tumor microenvironment confer resistance to cisplatin treatment in ovarian tumor cells by reducing the intracellular cisplatin accumulation. However, interferon (IFN)-γ derived from CD8^+^ T cells in the tumor stromal region attenuated platinum resistance through STAT1 phosphorylation and xCT downregulation in fibroblasts (**Figure 2**) (7). In contrast to the high expression of xCT in solid tumors, the xCT transporter is downregulated in hematologic malignancies such as chronic lymphocytic leukemia. Downregulation of xCT expression limits its ability to transport cystine for GSH synthesis (12). However, bone marrow stromal cells effectively import cystine, convert it to cysteine, and release it into the microenvironment for uptake by chronic lymphocytic leukemia cells to promote GSH synthesis (12). Interactions between stromal cells and leukemic cells are essential for the survival of chronic lymphocytic leukemia cells from drug-induced cytotoxicity (12). Furthermore, Cramer et al. reported that cysteinase, a glutathione inhibitor that degrades cysteine and cystine, suppressed the growth of prostate carcinoma allografts, reduced tumor growth in prostate and breast cancer xenografts due to depletion of intracellular GSH and consequent oxidative stress (13).

### GSH peroxidase 4 (GPX4)

The tripeptide GSH consists of glutamate, cysteine, and glycine and serves as a cysteine storage and transport form (14). It is synthesized by the consecutive action of two ATP-dependent enzymes, γ-glutamylcysteine synthetase, and GSH synthetase. The extracellular enzyme γ-glutamyltranspeptidase cleaves the γ-peptide linkage of GSH to release the product cysteinyl-glycine and glutamate. Cysteinyl-glycine can be hydrolyzed to cysteine and glycine, which can be imported into the cell (15, 16). Ferroptosis is an iron-dependent nonapoptotic cell death driven by lipid peroxidation and membrane damage, and is associated with a redox imbalance (17). GSH is an essential cofactor for GPX4 enzymatic activity (18). GPX4 converts GSH into oxidized glutathione (GSSG), which then reduces cytotoxic lipid peroxides to lipid alcohols. Inhibition of GPX4 activity leads to the accumulation of lipid peroxides, which triggers ferroptic cell death (19). When the cells encounter a decrease in cysteine levels, ferroptosis is triggered by the degradation of GPX4 *via* chaperone-mediated autophagy (20). Recently, Zhang et al. reported that cysteine starvation impairs GPX4 protein expression by inactivating mTORC1/4E-BP1-mediated protein translation. mTORC1 inhibitors also sensitize cancer cells to ferroptosis induced by GPX4 inhibitor in renal cancer UMRC6 cells (21). In addition, under dual depletion of cystine and GSH, hepatocellular carcinoma HepG2 cells undergo ferroptosis, characterized by a marked increase in lipid peroxidation, and this cell death program can be rescued by a ferroptosis inhibitor treatment (22). Moreover, Yang et al. reported that the administration of inhibitors targeting GPX4 directly or indirectly through GSH depletion suppresses tumor formation in mice bearing human fibrosarcoma HT-1080 xenografts (19). Fu et al. demonstrated that cisplatin-resistant gastric cancer cells had lower levels of ATF3 than their parental cells, which results in reduced ferroptosis due to low ROS, lipid peroxidation, and higher intracellular GSH levels (23). ATF3 sensitizes gastric carcinoma cells to cisplatin through ferroptosis induction by blocking the Nrf2/Keap1/xCT signaling pathway (23). In line with this notion, Fan et al. reported that the activation of the Nrf2/Keap1 pathway increased the xCT expression and diminished ferroptosis, which facilitating glioma cell growth (24). Also, Roh et al. showed that the inhibition of xCT sensitized cisplatin-resistant head and neck cancer (HNC) cell lines to cisplatin by inducing ferroptotic cell death *via* glutathione depletion and ROS accumulation (25).

### Hydrogen sulfide

H_2_S is produced in mammalian cells by three enzymes, including cystathionine β-synthase (CBS), cystathionine γ-lyase (CSE), and 3-mercaptopyruvate sulfurtansferase (3MST) in mammalian cells. CBS and CSE are located in the cytosol of cells, whereas 3MST primarily resides in and generates H_2_S in mitochondria. Studies have reported that of H_2_S involved in both the inhibition and advancement of cancer. Knockdown of CBS decreases bioenergetics, actions such as oxygen consumption and ATP production in colon and ovarian cancer cells (26, 27). H_2_S might enhance glucose uptake by stimulating GLUT activity, thereby accelerating glycolysis. This supports the production of intracellular ATP required by cancer cell proliferation (28). In addition, vascular endothelial growth factor increases H_2_S level by upregulating CSE expression in endothelial cells, thereby promoting angiogenesis of endothelial cells obtained from breast carcinomas (B-TECs) (29). It also prompts to deliver nutrients and oxygen to cancer cells (29). CSE knockdown suppresses vascular endothelial growth factor--induced migration of B-TECs (29). H_2_S can mediate hypoxia-induced angiogenesis in cancer progression by inhibiting the catabolism of H_2_S and increases the expression of CSE (30, 31). Moreover, H_2_S exerts a protective effect against various apoptotic stimuli through the activation of NF-κB and Nrf2 mediated by H_2_S-linked persulfidation (32, 33). In the same context, H_2_S is able to accelerate the cell cycle in cancer cells by upregulating the expression of proliferating cell nuclear antigen and cyclin-dependent kinase 4, thereby promoting cell proliferation in oral squamous cell carcinoma (**Figure 2**) (34).

### Taurine

Cysteine dioxygenases catalyzes the oxidation of cysteine to cysteine sulfinate. Cysteine sulfinic acid decarboxylase catalyzes the reaction, and carboxyl groups are removed to form hypotaurine, which subsequently generates taurine (35). Taurine, also known as 2-aminoethanesulfonic acid, is the most abundant free sulfur-containing amino acid in mammalian tissues, and possesses anti-oxidative, anti-inflammatory, and anti-apoptotic effects (36). Marcinkiewicz and Kontny reported that taurine has cytoprotection function and maintains the homeostasis of cells involved in acute and chronic inflammatory/oxidative stress (37). Several studies have confirmed that taurine displays a strong growth-inhibitory effect on various cancer types including colon cancer (38), lung cancer (39), hepatocarcinoma (40), melanoma (41), and breast cancer (42). El Agouza et al. reported that serum taurine levels decreased in patients with high breast cancer risk, which was linked to decreased angiogenesis (43). Moreover, taurine exerts a strong antitumor effect on rats harboring mammary carcinogenesis, which can be attributed to disturbances in the energy metabolism. Plasma concentrations of fumarate, malate, citrate, α-ketoglutarate, and pyruvate involved in glycolysis and the tricarboxylic acid (TCA) cycle are lower in the taurine-supplemented breast cancer mice group than their concentrations in a normally matched group of mice (44).

## Cysteine in carbon metabolism reprogramming

Metabolic reprogramming, an important cancer hallmark, refers to the ability of cancer cells to modify their metabolism, to support the increased energy demand due to the continuous growth and rapid proliferation of cancerous cells. In fact, the metabolic changes in glucose, lipids, and amino acids provide the cancer cells with the energy and substances needed for biosynthesis and the maintenance of biological functions (45). Amino acids participate in important processes such as oncogenesis and progression and are important raw materials for cell anabolism. Recently, Nunes et al. reported that cysteine promotes sulfur and carbon metabolic reprogramming, the underlying the adaptation of ovarian cancer cells to hypoxic microenvironment. (46). In fact, the xCT transporter localizes in mitochondria of ovarian cancer cells, and then an increase in intracellular cysteine facilitates ATP production under hypoxia conditions (46). Nunes et al. reported that cysteine allows ovarian cancer cells to adapt to hypoxic environments and to escape from carboplatin cytotoxicity (47, 48). Although it plays a minor role, transsulfuration contributes to *de novo* cysteine synthesis from methionine which is recognized as an additional mechanism for maintaining cysteine pools in the tumor microenvironment (49). Enhanced transsulfuration activity driven by the glycine *N*-methyltransferase may contribute to cysteine biosynthesis and promote cancer cell survival in cysteine-limited microenvironment, which has been demonstrated to support tumor growth *in vivo* (50). Liu et al. reported that upregulated transsulfuration pathway for cysteine synthesis in erastin-resistant ovarian cancer cells compensates for cysteine deprivation by xCT blockage, which is mediated by Nrf2-mediated CBS activation (51). Knockdown of CBS promotes cellular oxidative stress and lipid peroxidation, thus enhancing ferroptosis susceptibility (51). Moreover, patient-derived basal-like breast cancer tumors exhibited elevated expression of CBS. Anti-proliferative effect and diminished malignant transformation are observed after CBS silencing in basal-like breast cancer cells. Disruption of CBS inhibits both hypoxic response and tumor angiogenesis in basal-like breast cancer cell-derived xenograft tumors, which have larger intratumoral necrotic areas. It is due to the increased vulnerability to oxidative stress and ferroptosis induced by cysteine deprivation (52). Floros et al. reported that MYCN stimulates the transsulfuration pathway through the induction of the key enzymes CBS and methylthioadenosine phosphorylase, and further protects neuroblastomas from ferroptotic cell death (53). Cysteine is a valuable carbon source, since its catabolism produces organic compounds such as pyruvate, α-ketobutyrate, glutamate, serine, propionyl-CoA, succinate, and acetyl-CoA which supply the TCA cycle, and are intermediates for fatty acid synthesis (54, 55).

## Post-translational modifications targeting cysteine of protein in cancer

PTMs is a biochemical modification occurring to one or more amino acids on a protein during or after protein translation. PTMs are critical molecular events in a series of biological processes such as cell growth, proliferation, differentiation, metabolism, and apoptosis (56). In most proteomes, cysteine residues are frequently low but with high chemical reactivity. The cysteine thiols could be the nucleophilic residue attacking the substrate. Cysteine residues also react with each other to form disulfide bonds which stabilize the three-dimensional structure and alter the redox state (57). Protein oxidation, lipidation, and metabolites-mediated protein modification occur in cysteine (58). These PTMs are involve in various pathological events or diseases such as inflammation, carcinogenesis, aging, and neurodegenerative disorders (59–61).

### Protein oxidation

Cysteine residues in proteins are easily oxidized by ROS, reactive nitrogen species, reactive sulfur species, or GSH. Protein *S*-nitrosylation, the covalent attachment of nitric oxide (NO) moiety to the reactive thiol group of a cysteine residue to form *S*-nitrosothiol is an important PTM for most classes of proteins (62). Numerous *S*-nitrosylated proteins such as Bcl-2, p53, HIF-1α, PTEN, and Src are involved in cell survival, angiogenesis, tumorigenesis, and response to cancer treatment (63–68). NO impairs the apoptotic function of cells and increases resistance to cisplatin-induced cell death in human lung carcinoma cells. NO production induces *S*-nitrosylation of Bcl-2, which inhibits its ubiquitination and subsequent proteasomal degradation (64). In addition, *S*-nitrosylation on Cys498 residue of Src kinase induces autophosphorylation, which promotes nitric oxide-mediated cell invasion and resistance to anoikis in cancer cells (68, 69). NO donors such as sodium nitroprusside and *S*-nitrosoglutathione promote *S*-nitrosylation at the Cys183 residue of extracellular signal-regulated kinase 1/2 (ERK1/2), which leads to inducing apoptosis in U251 glioma cells (70). Therefore, *S*-nitrosylation affects a variety of proteins that play important roles in the cellular dysfunctions and contribute to cancer progression and response to chemotherapy.

### Protein lipidation

Protein lipidation is an important PTM that can reversibly or irreversibly attach lipid types to proteins, and the three major lipidation processes are palmitoylation, myristoylation, and farnesylation. Palmitoylated proteins are modified by the attachment of fatty acid to cysteine residues *via* thioester linkage. Palmitoylation of protein regulates its binding at the plasma membrane, lipid raft localization, and protein stability (71). In prostate and breast cancer cells, palmitoylation occurs at the cysteine residue 797 of the epidermal growth factor receptor (EGFR) residing in mitochondria, which stimulates the activation of EGFR. It promotes mitochondrial fusion and cell survival by upregulating mitochondrial prohibitin 2 and optic atrophy 1 protein levels (72). In addition, the isoprenyl group can react with cysteine thiol and bind to proteins, thus forming irreversible *S*-prenylation reactions such as farnesylation. Farnesyltransferase is an enzyme for farnesylation on the cysteine residue of the CAAX motif region of cytosolic RAS protein, resulting in RAS protein association with cellular membranes. Fatty acid synthase is a metabolic enzyme involved in liponeogenesis and its overexpression has been associated with poor prognosis and shorter disease-free survival in patients with prostate cancer, lung cancer, and sarcoma (73–75). Scribble (Scrib) organizes cell polarity gradients and suppresses aberrant growth signals in various human cancers. Scrib Cys4 and Cys10 residues are required for palmitoylation of Scrib by ZDHHC protein acyl transferases (76). The expression of the epithelial-mesenchymal transition transcription factor Snail leads to Scrib displacement from the plasma membrane to the cytosol, which is associated with disrupting *S*-palmitoylation of Scrib in epithelial cancer cells (77).

### Metabolite-mediated PTMs

**Figure 3:**
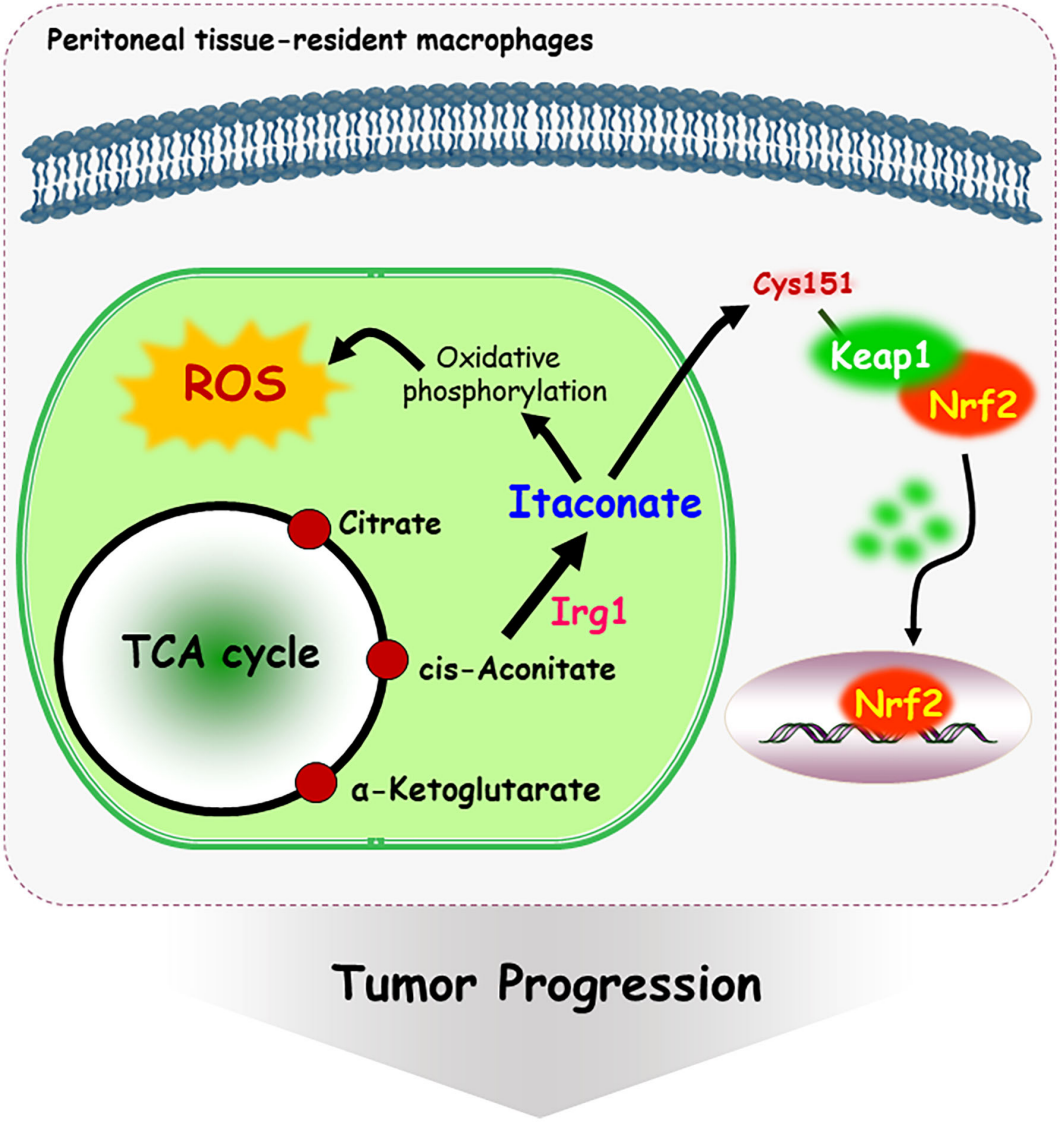
Mechanism of itaconate-mediated cysteine modification. Immune-responsive gene 1 (Irg1) is a mitochondrial enzyme-producing itaconate. Itaconate, covalently modifies at the Cys 151 residue of Keap1, following Nrf2 nuclear localization and activation. In addition, itaconate promotes tumor growth *via* oxidative phosphorylation-driven ROS generation in peritoneal tissue-resident macrophages.

Metabolites have been shown to be involved in critical biological changes and regulations. Itaconate, derived from citrate produced in the TCA cycle, contains α,β-unsaturated carboxylic acid, and covalently modifies at the Cys151 residue of Keap1 (78). Itaconate promotes tumor growth *via* oxidative phosphorylation-driven ROS generation in peritoneal tissue-resident macrophages and concomitant ROS-mediated mitogen-activated protein kinases (MAPKs) activation in tumor cells (79). Interestingly, *Nrf2* expression is significantly downregulated in peritoneal tissue-resident macrophages isolated from Immune-responsive gene 1 (*Irg1*) shRNA-injected tumor-bearing mouse (79). Irg1 is a mitochondrial enzyme that produces itaconates. Cys151 residue is required to inhibit Keap1-mediated Nrf2 degradation, thus itaconate upregulates Nrf2 levels *via* Keap1 alkylation (**Figure 3**). Moreover, 4-hydroxy-2-nonenal (HNE), a major α,β-unsaturated aldehyde product of n-6 fatty acid oxidation, is involved in metabolic and neurodegenerative diseases, inflammatory diseases, and cancer (80). 4-HNE can form adducts with Fas, a death receptor protein with a cysteine-rich extracellular domain (81). Also, 4-HNE induces Daxx protein level and promotes the export of Daxx from the nucleus to the cytosol in Jurkat T lymphocyte cells. Daxx is then bound to Fas, which leads to suppression of apoptosis (81). Moreover, cytoplasmic translocation of Daxx induces up-regulation of heat shock factor 1 associated stress-responsive genes, which may contribute to resistance to apoptosis.

## Role of cysteine residues for inhibitors targeting protein kinases and phosphatases

PTMs can control the biological function of numerous phosphatases, kinases, and transcription factors, which can alter the intracellular localization of target proteins or their interaction with binding partner proteins. Several studies have been conducted on cysteine modification by various endogenous and exogenous molecules/chemicals in various transcription factors (82). Recently, the ability of oxidative stress and small molecule inhibitor-induced cysteine modifications have received considerable attention for regulating the function of both protein phosphatases and kinases (83).

Protein phosphatases belong to two major families of phosphatases: serine/threonine protein phosphatases and protein tyrosine phosphatases (PTPs). PTPs regulate signal transduction pathways involving tyrosine phosphorylation and have been implicated in the development of cancer (84, 85). The active site of most classical PTPs is formed by the P-loop, which contains the conserved PTP signature motif (H/V)C(X)_5_R(S/T). The cellular redox state is involved in regulating tyrosine phosphatase activity through the reversible oxidation of catalytic cysteine to sulfenic/sulfinic acid (86). The catalytic cysteine is highly susceptible to oxidation and nitrosylation, leading to reversible or irreversible modifications that abolish its nucleophilic function and inactivate its enzyme activity (87, 88). Oxidation of the Cys residue in the active site of PTPs by ROS abrogates its nucleophilic properties, thereby inhibiting PTP activity (87). PTP1B acts as a negative regulator of multiple receptor tyrosine kinases, including the EGFR. NADPH oxidase 4 (NOX4)-mediated oxidation of PTP1B in the endoplasmic reticulum enhances EGFR phosphorylation (89). Bile acids can trigger mitochondrial ROS generation in hepatocytes that in turn act to mediate the inactivation of PTPs, resulting in the activation of EGFR (90). In addition, *S*-nitrosylation of the Cys215 residue by NO donors shields PTP1B from subsequent hydrogen peroxide-induced irreversible oxidation (88). PTPN22 (short for protein tyrosine phosphatase non-receptor type 22), known as a risk factor in multiple autoimmune disorders, reduces T-cell activity by removing phosphate groups from phosphorylated proteins such as LCK, Fyn, and Zap70, which are associated with T-cell receptor signaling pathway. Interaction between the non-catalytic cysteine at position 129 and the catalytic cysteine 227 shows the formation of a disulfide bond, which maintains its catalytic activity (91, 92). However, when Cys 129 residue of PTPN22 is mutated, the disulfide bond cannot form and the enzyme is exposed by oxidation, resulting in inactivation (93).

Recently, the discovery of reversible/irreversible covalent inhibitors targeting cysteine residues in and around the ATP-binding pocket of kinases has been gaining considerable attention (94). Hypothemycin, one of the resorcylic acid lactones, is a representative inhibitor targeting cysteine residues located in the ATP site of Ser/Thr/Tyr protein kinases. The α,β-unsaturated enone moiety of resorcylic acid lactones is susceptible to Michael addition reaction with a conserved cysteine residue (Cys166 in human ERK2) (95). In addition, hypothemycin inhibits the phosphorylation of the mitogen-activated protein kinase kinase (MEK)3/6 substrate p38, the MEK4/7 substrate c-Jun N-terminal kinase (JNK), and the TGF-β-activated kinase 1 (TAK1) substrate IκB kinase β. MEK and TAK1 contain the conserved cysteine residue corresponding to ERK2 (95). Afatinib, the first covalent inhibitor of EGFR approved by the FDA, binds to Cys797 residue on the kinase domain of EGFR in the “DFG-in” conformation (94). Tan et al., reported that FIIN-2, an irreversible covalent FGFR inhibitor, formed the covalent binding mode at the Cys477 residue of FGFR4 in the “DGF-out” conformation (96). Furthermore, a newly synthesized compound targeting Cys174 at the DFG-1 position in TAK1 is considered a type II inhibitor (97). There is a growing interest in the discovery of kinase inhibitors that can be reversibly or irreversibly modulated by targeting cysteine. Many researchers have focused on understanding the mode of action of inhibitors that can act differently depending on the DGF-in or DFG-out conformation.

## Conclusion and future perspectives

**Figure 4:**
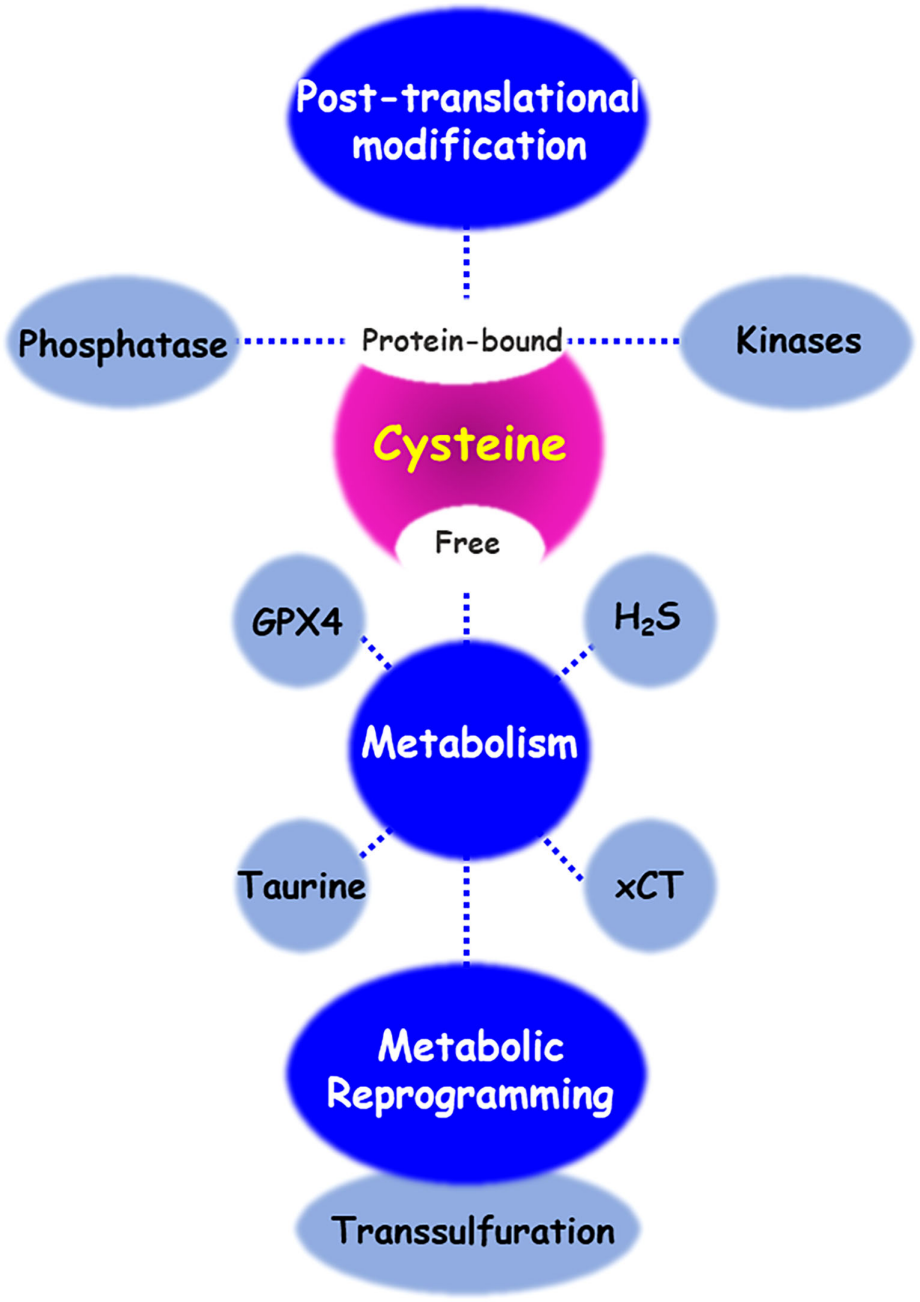
Cysteine metabolic fate. There are diverse regulatory mechanisms of cysteine bound to or free from proteins. Enzymes and metabolites of cysteine transport and metabolism enables cancer cells to contributing cancer progression and acquiring chemoresistance. In addition, cysteine residue of proteins undergoes various post-translational modifications, to maintain its redox homeostasis. Oxidative stress and small molecule inhibitors-induced cysteine modifications regulate the function of both protein phosphatases and kinases.

As shown in the **Figure 4**, cysteine has different fates including the synthesis of cysteine-derived molecule, sulfur/carbon metabolic reprogramming, and venue for post-translational protein modification of various proteins or discovery of drug inhibitors. Intracellular and extracellular alteration of amino acid metabolism in the tumor microenvironment can influence cancer growth, progression, and metastasis. Cysteine, as a multifaceted precursor, plays a central role in cellular metabolism and contributes to the survival and proliferation of cancer cells. Changes in the redox state of cells by cystine/cysteine circuitry control the ROS levels, which modulates cellular signal transduction pathways involved in cell survival and resistance to chemotherapy. Several proteins, involving covalent or non-covalent cysteine modifications have been also identified. Naturally or synthetic chemotherapeutic agents exert their effects through oxidation or modification of cysteine thiol groups present in the cellular signal molecules mediated by phosphatases and kinases. Covalent or non-covalent inhibitors are again attracting attention, and they target site-specifically a cysteine residue near the active pocket of druggable proteins. Further studies are needed to validate the reversible/irreversible modes of action of covalent inhibitors.

## Author contributions

J-YM documented paper, drew figures, and summarized data. K-SC and D-HK designed paper and supervised the manuscript. All authors contributed to the article and approved the submitted version.
